# Mapping human papillomavirus, Epstein–Barr virus, cytomegalovirus, adenovirus, and p16 in laryngeal cancer

**DOI:** 10.1007/s12672-022-00475-4

**Published:** 2022-03-21

**Authors:** Alexandra Schindele, Anna Holm, Karin Nylander, Annika Allard, Katarina Olofsson

**Affiliations:** 1 Department of Clinical Sciences, Otorhinolaryngology, ÖNH-Kliniken Östersunds Sjukhus, Umeå University, 831 83 Östersund, Sweden; 2 Department of Medical Biosciences, Pathology, Umeå University, Umeå, Sweden; 3 Department of Clinical Microbiology, Clinical Virology, Umeå University, Umeå, Sweden; 4 Department of Clinical Sciences, Otorhinolaryngology, Umeå University, Umeå, Sweden

## Abstract

**Purpose:**

Apart from tobacco and alcohol, viral infections are proposed as risk factors for laryngeal cancer. The occurrence of oncogenic viruses including human papilloma virus (HPV) and Epstein–Barr virus (EBV), in laryngeal squamous cell carcinoma (LSCC) varies in the world. Carcinogenesis is a multi-step process, and the role of viruses in LSCC progression has not been clarified. We aimed to analyze the presence and co-expression of HPV, EBV, human cytomegalovirus (HCMV) and human adenovirus (HAdV) in LSCC. We also investigated if p16 can act as surrogate marker for HPV in LSCC.

**Methods:**

Combined PCR/microarrays (PapilloCheck®) were used for detection and genotyping of HPV DNA, real-time PCR for EBV, HCMV and HAdV DNA detection, and EBER in situ hybridization (EBER-ISH) for EBV detection in tissue from 78 LSCC patients. Additionally, we analyzed p16 expression with immunohistochemistry.

**Results:**

Thirty-three percent (26/78) of LSCC tumor samples were EBV positive, 9% (7/78) HCMV positive and 4% (3/78) HAdV positive. Due to DNA fragmentation, 45 samples could not be analyzed with PapilloCheck®; 9% of the remaining (3/33) were high-risk HPV16 positive and also over-expressed p16. A total of 14% (11/78) of the samples over-expressed p16.

**Conclusion:**

These findings present a mapping of HPV, EBV, HCMV and HAdV, including the HPV surrogate marker p16, in LSCC in this cohort. Except for EBV, which was detected in a third of the samples, data show viral infection to be uncommon, and that p16 does not appear to be a specific surrogate marker for high-risk HPV infection in LSCC.

**Supplementary Information:**

The online version contains supplementary material available at 10.1007/s12672-022-00475-4.

## Introduction

Laryngeal cancer is detrimental for voice and airway, leading to life-changing consequences.

The worldwide annual incidence of newly detected laryngeal cancer is 210,000 cases/year. The estimated prevalence is ∼1,000,000 cases/year and 126,000 deaths/year [1].

Laryngeal squamous cell carcinoma (LSCC) can develop from several causes, with tobacco and alcohol use being recognized risk factors [2]. Other proposed risk factors are viral infections. Both human papillomavirus (HPV) and Epstein–Barr virus (EBV) are associated with cancer in humans, including in the upper airway [3].

EBV and HPV can each lead to cancer development independently, but these viruses can also co-infect and enhance cancer progression through interactions with other mechanisms [3].

Cancer in the tonsils and base of tongue is associated with high-risk HPV genotypes HPV16 and HPV18. These types of cancer differ from non-HPV associated malignancies both biologically and clinically [4], which has led to adjustments in tumor classification.

p16 is the second most common tumor suppressor gene [5], and its protein expression has been shown to be a reliable surrogate marker for HPV-infection in tonsillar cancer [6], whereas expression of p16 in mobile tongue cancer has no demonstrated correlation with HPV-infection [7].

The presence of high-risk HPV types in LSCC has been reported in 5–24% of cases [8]. Variations in HPV types and reporting are due to regional differences related to exposure as well as the use of different methods for identifying HPV types.

EBV is reported in 60% of laryngeal cancers [9]. EBV is well established as a factor in B cell and epithelial malignancies, encoding oncogenes such as EBV encoded nuclear antigens, (EBNA 1–3) and LMP1-2. In nasopharyngeal cancer type 2 and 3, and in oral cancer, EBV has an established contributing role [3]. EBV downregulates p16 expression by suppressing the p16 gene promotor via latent membrane protein 1 (LMP-1) [5].

EBV, HPV, human cytomegalovirus (HCMV), and human adenovirus (HAdV) all replicate in the airway epithelium. They are transmitted via saliva or inhalation of droplets from infected individuals and can establish persistent infections in their host in part through evading host immune surveillance.

HCMV and HAdV infections can be devastating in immunocompromised patients. HCMV is not a typical human tumor virus, but is thought to have oncomodulatory activity [4]. HCMV and HAdV have molecular mechanisms that aid in cell transformation [10]. The common cold virus HAdV is not known to have oncogenic potential in humans, but its early gene products can transform rodent cells [11].

Carcinogenesis is a multistep process, and the role of viruses in this progression in LSCC has not been fully identified. If viruses turn out to be important in development of LSCC, anti-viral vaccines could potentially be used for prevention, and possibly even treatment of this disease. Vaccination holds a promise for a vast reduction of HPV-related cancer in tonsils and base of tongue. Our hypothesis is that LSCC is associated with oncogenic/oncomodulatory virus. Therefore, our purpose is to map HPV, EBV, HCMV and HAdV in a group of cases with LSCC, and study co-infection. In addition, we aim to analyze if p16 is a reliable surrogate marker for HPV in LSCC.

## Materials and methods

### Materials

We identified 236 consecutive LSCC samples from cases between 2000 and 2017 in SymPathy (Tieto, Finland). Slides were histopathologically re-examined (by KN and KO) for correct diagnosis and classification of cancer site.

Exclusion criteria for analysis were: (1) repeated biopsies from the same case (112 samples), (2) previous radiotherapy to the neck (39 samples), (3) classification errors (5 samples) and (4) too little tumor tissue for analysis (2 samples).

### Patient characteristics

SymPathy data provided access to clinical chart databases to supplement patient characteristics.

### Descriptive data presented in two groups

**Fig. 1:**
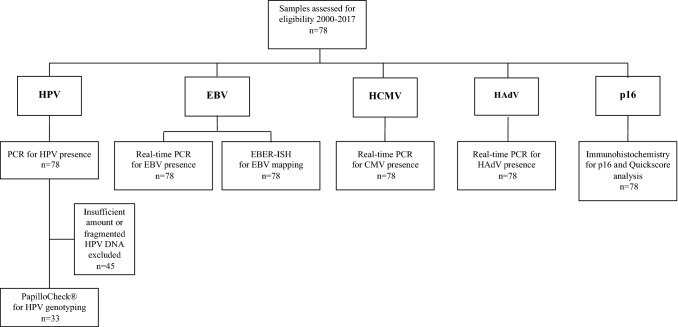
Flowchart of LSCC samples for virus and p16 analysis

Descriptive data on 78 LSCC samples included in the PCR analysis of EBV, HCMV, HAdV and p16 (analyzed by IHC) are presented. Due to HPV DNA fragmentation, 45 LSCC samples could not be analyzed, and were therefore drop-outs in the HPV PCR results (Fig. 1). To clarify the possible impact of the dropouts, we analyzed the dropout group separately in categories for age, sex, LSCC site (supraglottic, glottic, subglottic) and smoking status (non-smoker, former smoker, smoker) (Table 1).

**Table 1:** Case characteristics

Characteristics	Total group n = 78 (%)	HPV subgroup n = 33 (%)
*Age*		
Mean [SD]	68.1 [± 10.3]	67.2 [± 10.2]
Range	41–86	46–83
*Sex*		
Female	15 (19)	6 (18)
Male	63 (81)	27 (82)
*Site*		
Supraglottic	12 (15)	5 (15)
Glottic	64 (82)	27 (82)
Subglottic	2 (3)	1 (3)
*Smoking status*		
Non-smoker	14 (18)	6 (18)
Female	4 (5)	2 (6)
Male	10 (13)	4 (12)
Former smoker	21 (27)	8 (24)
Female	3 (4)	1 (3)
Male	18 (23)	7 (21)
Smoker	42 (54)	19 (58)
Female	7 (9)	3 (9)
Male	35 (45)	16 (49)
Smoking data missing	1 (1)	–

Results presented in numbers, and proportion (in brackets). *SD*  standard deviation presented in square brackets

### Cases

Seventy-eight (78) LSCC samples were assessed for eligibility. The case ages ranged from 41 to 86 with a mean of 68.1 years (± 10.3). The sex distribution was 15 (19%) women and 63 (81%) men, and other clinical characteristics are shown in Table 1.

### HPV subgroup

Thirty-three LSCC samples underwent HPV genotyping, with an age range of 46–83, mean of 67.2 years (± 10.2). The sex distribution was 6 (18%) women and 27 (82%) men.

### Methods

Combined PCR/microarrays (PapilloCheck®, Greiner BioOneGmbH, Frickenhausen, Germany) were used for detection and genotyping of HPV and real-time PCR for EBV, HCMV and HAdV DNA detection. EBER in situ hybridization (EBER-ISH) was used for EBV detection in slides from LSCC tumors. In addition, we used immunohistochemistry (IHC) for mapping of p16 (Fig. 1).

### PCR for HPV, EBV, HCMV and HAdV

DNA was prepared by cutting five 10‐μm sections from each LSCC biopsy followed by routine extraction using the QIAamp DNA FFPE Tissue Kit (QIAgen, Hilden, Germany). Quality and quantity of total DNA was measured spectrophotometrically.

HPV detection by PapilloCheck® requires a DNA fragment of 350 bp for successful PCR performance. To assess the degree of DNA fragmentation prior to detection and typing using the PapilloCheck®, we performed three different PCR assays amplifying amplicon lengths of 536, 268 or 100 bp of the ß-globin housekeeping genes, and one real-time PCR assay detecting 60 bp of the RNasP housekeeping gene.

The PapilloCheck® is a multiplex PCR method with fluorescent primers, for genotyping of 24 HPV-types including 15 high‐risk HPV types; HPV 16, 18, 31, 33, 35, 39, 45, 51, 52, 56, 58, 59, 68, 73 and 82, two probably high or intermediate risk types; HPV 53 and 66, and seven low‐risk HPV types; HPV 6, 11, 40, 42, 43, 44 and 70.

For EBV detection, a real-time PCR assay of the *Bam*HI-W region of the EBV genome was used. The sequences of the forward and reverse primers were 5′-GCATAATGGCGGACCTAGG-3′ and 5′-AAGATAGCAGCAGCGCAGC-3′, respectively, flanking the dual-labelled probe 5′-(FAM)TAAAACCCCCAGGAAGCGGGTCTATG(TAMRA)-3′. Real-time PCR detection towards the polymerase gene of HCMV consisted of the amplification primers 5′-TGDGTCACGCCGTTCTCTAG-3′ and a 50:50 mix of 5′-ATCATCATGGCCCACAACCT-3′ and 5′-ATCATCATGGCTCACAACCT-3′, flanking the dual-labelled fluorescent probe 5′-(FAM)ACTCGCCACCCGGCACCAAC(TAMRA)-3′.

For detection of HAdV DNA a real-time PCR assay of the hexon region was used. The sequences of the degenerated forward and reverse primers were 5-CWTACATGCACATCKCSGG-3′ and 5′-CRCGGGCRAAYTGCACCAG-3′, respectively, flanking the dual-labelled probe 5′-(FAM)CCGGGCTCAGGTACTCCGAGGCGTCCT (TAMRA)-3′.

### EBER-ISH for EBV mapping

The EBER-ISH is an in-situ hybridization kit for EBER transcripts (Epstein–Barr Encoding Region), and this was applied for EBV mapping in tumor and connective tissue.

Two tissue sections were cut from each LSCC biopsy, one was incubated with an RNA-positive control probe (800-2846, Ventana). The second section was tested for EBER-1 and EBER-2 by the Inform EBER Probe (800-2842, Ventana Medical Systems, Roche Diagnostics GmbH, Mannheim Germany). NBT- BCIP Detection System (800-092, Roche Diagnostics GmbH, Mannheim, Germany) was employed for visualization. We used a Ventana BenchMark ULTRA system (Roche Diagnostics AG, Switzerland) for staining.

EBER-positive cells were counted in tumor and connective tissue by three of the authors (AS, AH and KN). Consensus was reached after comparison of individual results.

EBER was graded as positive when there were ≥ 2 positive cells and graded as negative when there were one or no positive cells.

### p16 Immunohistochemistry (IHC) and Quickscore analysis

To detect the p16 protein, five µm sections were cut from paraffin blocks. The staining system (Ventana BenchMark ULTRA, Roche Diagnostics AG, Switzerland) was applied, and slides pretreated in CC1 (cell condition solution 1, pH 8.4; Ventana) for 48 min. For detection of p16, CINtec® p16 Histology (Roche Diagnostics AG, Switzerland) was used. To visualize the antibody, the Opti View DAB IHC Detection Kit (Ventana Medical Systems) was employed.

p16 expression was evaluated by three members of the group (AS, AH and KN) using a light microscope (Nikon Eclipse Ci, Tokyo, Japan) and a semiquantitative Quickscore system by Detre et al. [12]. Using this system, we evaluated both proportion of p16 stained cells and the staining intensity (see Supplementary Information). Multiplying the scores for proportion of stained cells with intensity, results in a Quickscore ranging from 0 to 18. After comparison rounds consensus was reached.

### Statistics

Descriptive statistics (IBM SPSS USA, version 26) were presented regarding age, gender, smoking, cancer site and presence of virus and p16. Cross tabulations were used to describe numbers and proportions of virus and p16. The association between virus and p16 were analyzed using Chi-square test and Fisher’s exact test. A probability value of equal or less than 0.05 (2-sided) was considered statistically significant.

## Results

### HPV

HPV was assessed in a two-step fashion. First, 45 samples were excluded due to DNA fragmentation, exhibiting fragments smaller than the 350 bp needed for successful detection and typing. To ensure correct sorting, an HPV analysis with PapilloCheck® was performed on these excluded samples, without adding new results. Real-time PCR of the endogenous target gene RNAsP indicated that all samples contained DNA of at least 60 bp.

**Fig. 2:**
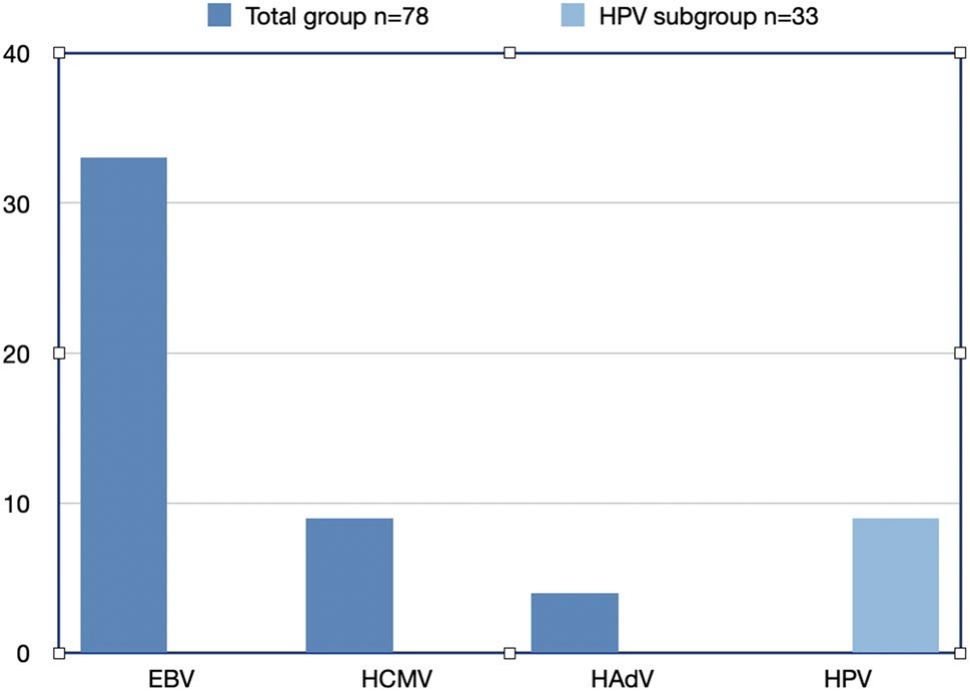
EBV, HCMV, HAdV in analyzed in 78 LSCC samples. HPV analyzed in subgroup with 33 samples, all samples were high-risk HPV16 positive. Results presented in percentage of positive samples

Secondly, we performed PapilloCheck® on a sub-group of 33 samples. Nine percent (3/33) were high-risk HPV16 positive (Fig. 2).

Two of three HPV16 positive cancers were in the true vocal cords, both patients were females and one also a smoker. The third patient was a non-smoking male with subglottic cancer. We did not identify any imbalance comparing the sub-group (n = 33) to the group in total (n = 78) regarding age, sex, site, or smoking status.

### HPV and co-infections

**Fig. 3:**
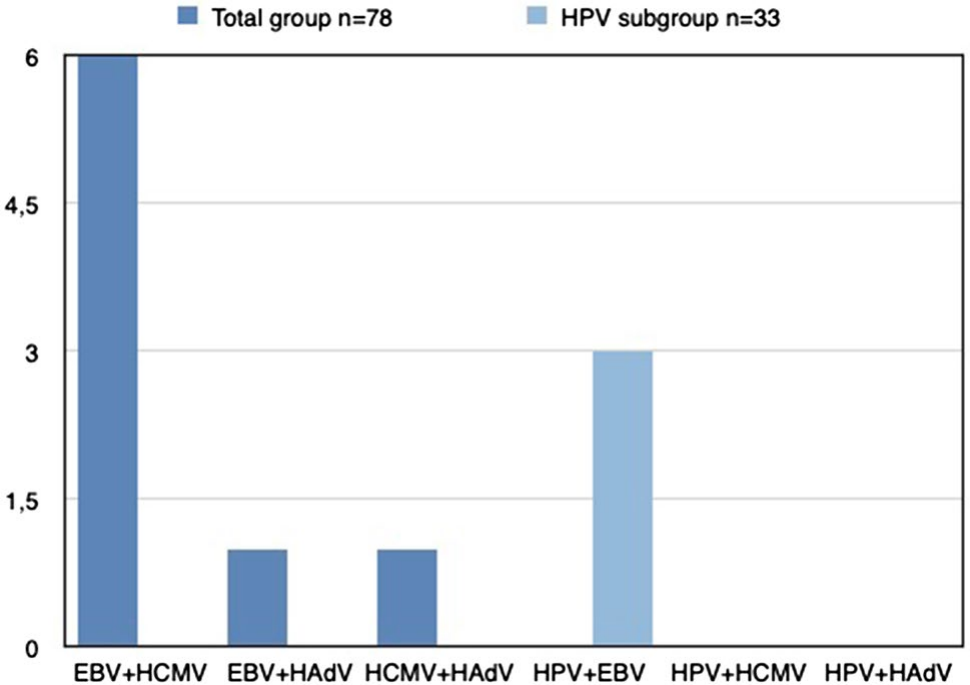
Viral co-infections in LSCC samples. Results presented in percentage of positive samples. None of the patients had infection with more than two viruses simultaneously

One patient was positive for both HPV and EBV (1/33) (Fig. 3), whereas 15/33 were EBV positive, but HPV negative.

### HPV and p16

The three HPV16 positive samples had a p16 QS of 12 (1) and 18 (2).

### EBV

**Fig. 4:**
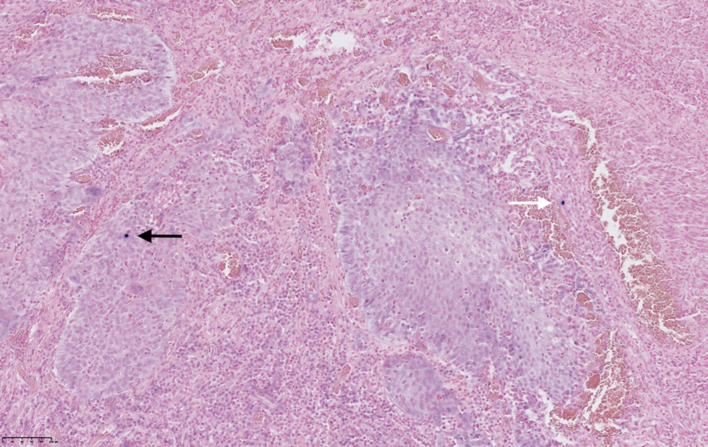
EBER in situ hybridization (EBER-ISH) applied for EBV mapping in tumor and connective tissue of a LSCC sample. Signals are detected as purple stained cells. EBER positive cells in tumor (black arrow) and connective tissue (white arrow)

We detected EBV by PCR in 33% (26/78), comprising tumor and connective tissue. Thereafter, EBER-ISH revealed distribution of EBER-positive cells in the tissue (Fig. 4).

The amount of positive cells/slide ranged from 2 to 10, with a mean of 5 cells in tumor tissue and 4.6 cells in connective tissue. Five percent (4/78) of samples were EBER-positive in tumor tissue, and 6% (5/78) were EBER-positive in connective tissue. Two of these samples were EBER-positive in both tumor and connective tissue, thus 9% (7/78) of LSCC samples were EBER-positive (Table 2).

**Table 2:** EBER-stained cells in tumor and/or connective tissue in LSCC samples

EBER-stained cells in LSCC samples	Positive (%)	Negative (%)
EBER-stained cells in tumor	4 (5)	74 (95)
EBER-stained cells in connective tissue	5 (6)	73 (94)
EBER in tumor + connective tissue	2 (3)	76 (97)
EBER-stained cells in total of 78 samples	7 (9)	71 (91)

Results presented in numbers and proportion (brackets)

### HCMV and HAdV

Nine percent (7/78) were HCMV positive and 4% (3/78) of samples were HAdV positive (Fig. 2).

### EBV, HCMV and HAdV co-infections

#### EBV and HCMV

Six percent (5/78) of samples were positive for both EBV (PCR) and HCMV (Fig. 3).

#### EBV and HAdV

One percent (1/78) of the samples were positive for both EBV and HAdV (Fig. 3).

#### CMV and HAdV

One percent (1/78) of the samples were positive for both HCMV and HAdV (Fig. 3).

### p16

**Fig. 5:**
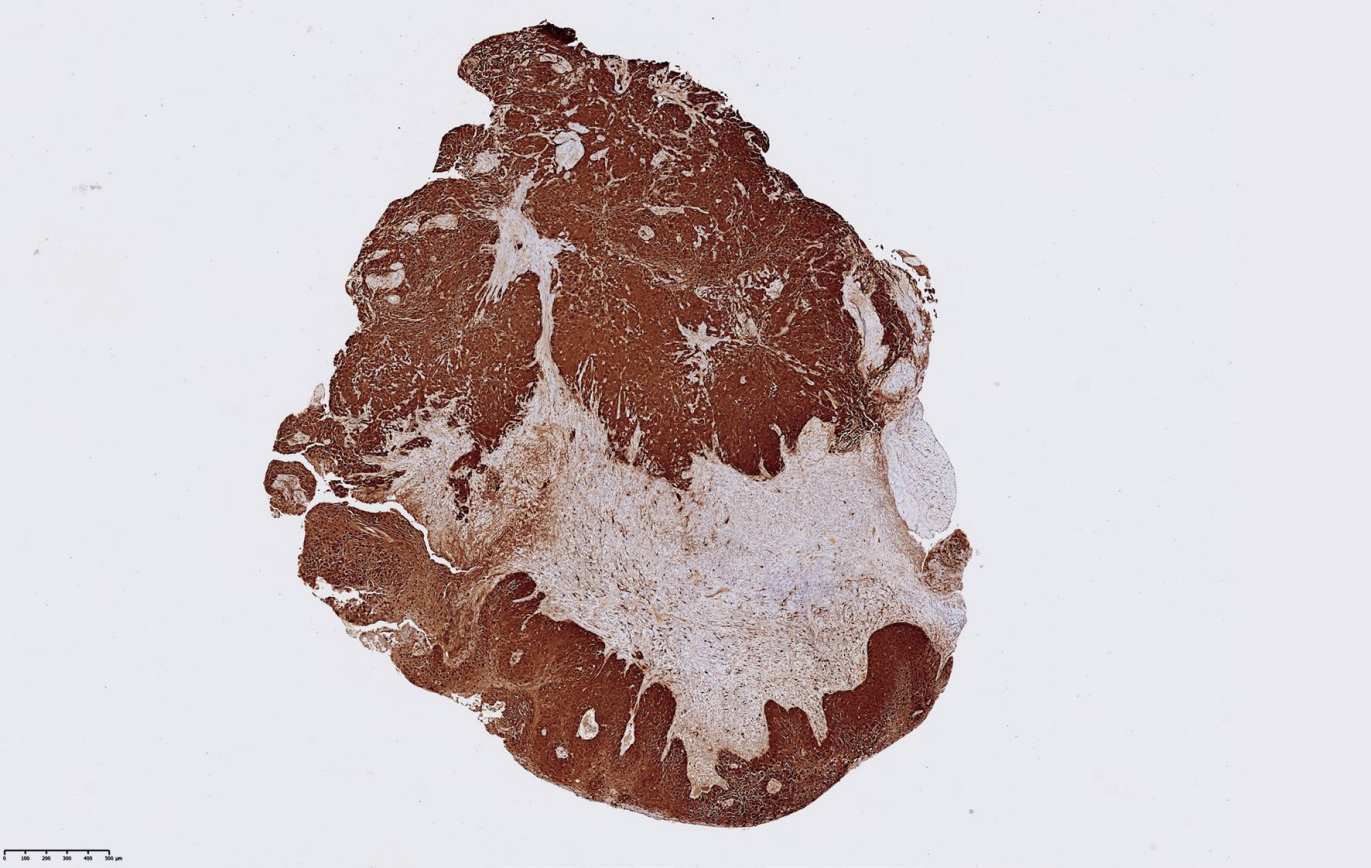
Representative p16 immunohistochemistry (IHC) staining in a LSCC sample with high Quickscore = 18 (proportion: 6 × intensity: 3)

The analysis of p16 in tumor tissue for intensity and proportion showed a Quickscore (QS) ranging from 0 to 18 (Fig. 5).

**Fig. 6:**
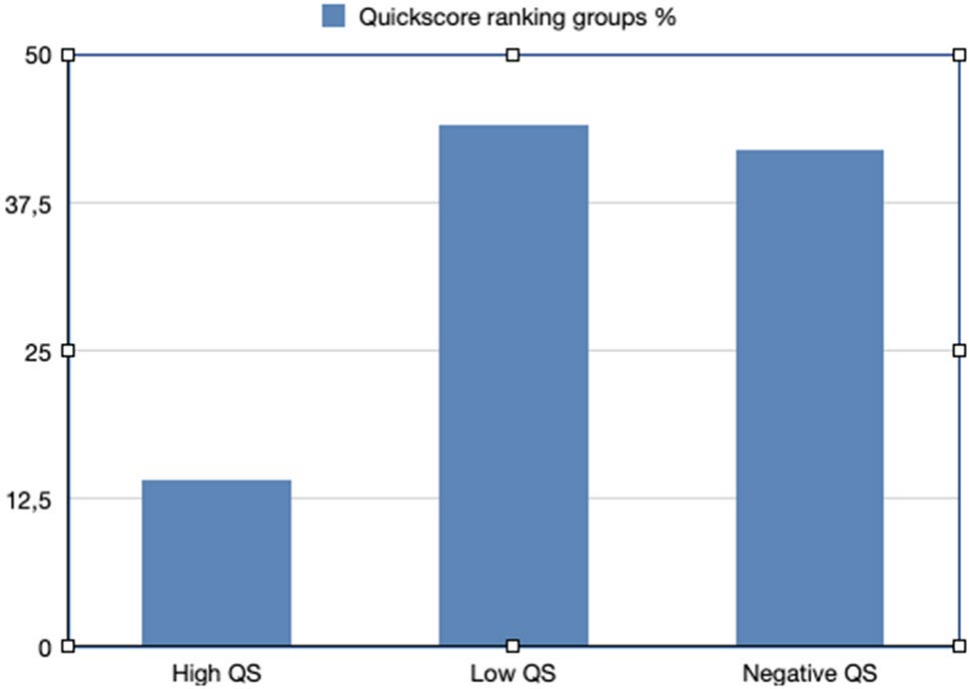
p16 Quickscore ranking groups presented in percentage

We ranked our Quickscore results in three groups: negative QS (QS = 0), low QS (QS = 1 and 2) and high QS (QS = 3–18). The results were: negative QS = 42% (33) of samples, low QS = 44% (34) and high QS 14% (11) of samples (Fig. 6). Nine percent (3/33) were HPV16 positive, and 58% (45/78) of LSCC samples expressed p16.

## Discussion

We performed a complete mapping of four different viruses and p16 in the same group of cases with LSCC, which showed that 33% (26/78) of samples were EBV infected and 9% (3/33) were high-risk HPV16 positive. These oncogenic viruses were expressed individually; in one case only (of 33) co-expression was noted.

Three out of 11 p16 high QS samples correlated with high-risk HPV16 positive samples, indicating that p16 was not a very sensitive surrogate marker for HPV16 in LSCC.

The common upper airway viruses HCMV and HAdV, were present in low amounts; 9% (7/78) were HCMV positive and 4% (3/78) HAdV positive. It would be relevant to map the same viruses in non-tumorous laryngeal mucosa, but ethical consideration makes it impossible to collect samples from a healthy control group, as the true vocal cords are susceptible to injuries in this type of sampling process.

### HPV

We searched for high- and low risk HPV types with PapilloCheck® in 33 LSCC samples. Nine percent (3/33) were high-risk HPV16 positive. Remaining 45 samples could not be analyzed due to random fragmentation of DNA caused by aging. Comparison of the groups did not show any differences regarding age, sex, site, or smoking status.

PapilloCheck® is a readily available, sensitive, and robust technique, used routinely in the clinical setting for screening of high and low- risk HPV types in the cervix. E6/E7 mRNA technique is by some considered a reference diagnostic test of active HPV infection [8, 13]. However, the commercially available E6/E7 mRNA tests have reduced capacity to detect multiple HPV genotypes.

Regardless of the detection method, HPV positivity appears to be low in LSCC. E6/E7 mRNA expression is reported between 2 and 8.6% [13, 14], and HPV DNA < 10% in LSCC [8]. This is coherent with our findings of 9% positive HPV16 in our Northern Swedish cohort.

### EBV

Using multidimensional outcome measures, we first found EBV by PCR in 33% of the tumor samples. Secondly, mapping of EBER-stained cells revealed roughly equal distribution in tumor (5%) and adjacent connective tissue (6%). EBER-ISH is not a quantitative measure, but it can map location of the EBV-infected cells in one slice of the total tissue sample. Epstein–Barr Encoded Regions 1 and 2 (EBERs) are good markers of EBV infection and present in numerous copies in latently EBV infected cells. They are easily identified by in situ hybridization (ISH) [15]. EBERs are thought to aid in the activation of oncogenic signaling pathways [16].

EBV PCR, on the contrary, was used to assess EBV presence or absence in a bigger piece (2 × 2 mm) of the tumor. This explains the different numeric results between PCR and EBER-ISH.

It is important to distinguish EBV in actual tumor epithelial cells vs non-tumor cells, since EBV infection of epithelial cells is considered pathological [17]. It is unclear if EBER in connective tissue is clinically significant, and analysis by PCR therefore implies the risk of detecting virus in the whole tissue sample, without being tumor specific. Whether our finding of EBER-stained cells in epithelial tumor tissue represents an oncogenic effect of EBERs in LSCC, can only be a matter of speculation. Nevertheless, EBV is well proposed as a causative agent for two epithelial malignancies in close proximity to the larynx, such as oral squamous cell carcinoma and nasopharyngeal carcinoma [18]. The epithelial cancer rising from the nasopharyngeal mucosal lining is predominantly associated with EBV-infection, especially the non-keratinizing subtypes (2 and 3), most common in endemic areas, such as southern China [19].

### HPV and EBV co-infection

We found one patient (1/33) positive for both HPV and EBV in LSCC samples. EBV is known to interact with HPV in adult-onset recurrent respiratory papillomatosis, a benign lesion on the vocal folds with a risk of malignant conversion [20]. In LSCC in a Polish population, co-infection with HPV (36%), EBV (60%) and HCMV (12%) was reported [9]. Furthermore, Jiang et al. found co-infection of HPV and EBV in tonsillar (25%) and base of tongue (70%) cancer [21]. However, it is still not certain whether simultaneous HPV and EBV presence in carcinoma tissue of the upper airway simply reflects a coincidental infection or has an additive effect on carcinogenesis.

### HCMV and HAdV

One of the target viruses in the analysis, HCMV, might have “oncomodulatory” activities, resulting in secretion of cellular and viral growth factors and cytokines in tumor microenvironment [3]. Nine percent (7/78) were HCMV positive, and 6% (5/78) both HCMV and EBV positive. Co-infection with multiple herpesviruses is common in both immunocompetent and immunocompromised patients with respiratory infections. Particularly the combination EBV (30%) and HCMV (25%) in the respiratory tract, is prevalent in immunocompromised adults [22]. Thus, the importance of EBV and HCMV co-infection in laryngeal cancer is of uncertain significance.

None of the LSCC samples was HCMV and HPV positive. Although implicated as a cofactor in HPV-mediated carcinogenesis, an association between oral cancer and HCMV has not yet been identified [23].

The second target virus, HAdV, confers mild and self-limiting infections such as the “common cold”. HAdV is oncogenic in rodents, but oncogenicity has not been observed in humans [24]. Humans can mount an immune response against HAdV-transformed cells [10]. Data showed 4% HAdV positivity. Two samples were co-infected with HAdV and EBV or HCMV.

### p16

There is not a widely accepted specific p16 cut-off value for positivity in the literature. The College of American Pathologists recommends that for positive diagnosis, p16 shows strong, diffuse nuclear and cytoplasmic staining in 70% of tumor cells [25].

We used the Quickscore (QS) method from Detre et al. [12] to assess p16 expression in tissue sections. This method takes into account the proportion of positively stained cells and multiplies it with the intensity of the staining. High p16 QS, defined as ≥ 12, corresponds to 80–100% of tumor cells expressing p16, fulfilling the requirements of the above-mentioned guidelines.

The tumor suppressor protein p16 was expressed with high intensity and quantity in the HPV16 positive cancer samples, whereas 8 samples over-expressed p16 without any correlation to HPV16. This supports the interpretation that p16 is not a reliable surrogate marker for HPV infection in LSCC.

### Oncogenicity

Whether HPV, EBV, HCMV and HAdV are just ‘passerby’ viruses in LSCC, or have oncogenic or oncomodulatory capacities, is a relevant clinical question. HPV and EBV have capabilities of virus-induced metabolic changes that share similarities with tumor associated metabolic reprogramming [10]. Metabolic reprogramming is probably an important feature of viral oncogenesis, together with modulation of cell cycle progression, resistance to cell death and evasion from immune surveillance. HCMV and HAdV, on the other hand, are upper airway viruses which may catalyze oncomodulatory effects, and are therefore relevant to study in LSCC.

## Conclusion

This is the first thorough report mapping HPV, EBV, HCMV and HAdV, including p16, in the same group of patients with LSCC. Except for EBV, which was detected in a third of the LSCC samples, viruses were not found in a large proportion of cases with this tumor type in this cohort. The findings also demonstrate that p16 may not be reliable as a surrogate marker for high-risk HPV infection in LSCC.

## Supplementary Information

Supplementary file 1 Quickscore system by Detre et al. Figure illustration by PhD Anna Holm (DOCX 28 KB)

## Data Availability

The data that support the findings of this study are available on request from the corresponding author.
