# Supplementary material for: Mapping human papillomavirus, Epstein–Barr virus, cytomegalovirus, adenovirus, and p16 in laryngeal cancer
Source: Discov Oncol. 2022 Mar 21;13:18. doi: 10.1007/s12672-022-00475-4 (PMC8938541; doi:10.1007/s12672-022-00475-4)
Supplement: Supplementary file 1 — Quickscore system by Detre et al. Figure illustration by PhD Anna Holm (DOCX 28 KB) [file 12672_2022_475_MOESM1_ESM.docx]

***Supplementary Information*** Quickscore system by Detre et al. Figure illustration by PhD Anna Holm

Proportion:

1. 0-4%
2. 5-19%
3. 20-39%
4. 40-59%
5. 60-79%
6. 80-100%

Intensity:

1. Negative
2. Weak
3. Intermediate
4. strong

Quickscore with a range from 0 to 18

=

x
